# Stomach ulcer caused by mistakenly oral medication of 14,400 mg ibuprofen: A case report

**DOI:** 10.1097/MD.0000000000033812

**Published:** 2023-05-17

**Authors:** Zhang Meijuan, Penglong Yu, Jie Yuan, Tao Yu, Dan SUN

**Affiliations:** a Shaanxi Provincial Hospital of Traditional Chinese Medicine, Xi’an, Shaanxi, P.R. China; b Chongqing Three Gorges Medical College, Chong Qing Shi, Feng Jie Xian, P.R. China.

**Keywords:** epigastric pain, housewife, hypertension, ibuprofen sustained-release CA, peptic ulcer

## Abstract

Gastric mucosal ulcer caused by drug overdose is very rare in clinical practice, and here is a case of gastric antral ulcer caused by drug overdose.

**Patient concerns:**

A 35-year-old housewife from a mountainous region in China took 48 Ibuprofen Sustained-Release capsules (300 mg/capsule) orally at 1 time. Because of severe tingling in the upper abdomen accompanied by a sharp increase in blood pressure, she came to the doctor 48 hours later.

**Diagnoses:**

Gastric antral ulcer (multiple stage A1), duodenococcitis, chronic nonatrophic gastritis, Helicobacter pylori infection, moderate depression, and cognitive impairment.

**Interventions:**

Acid suppression, antihypertensive and a series of symptomatic treatments.

**Outcomes:**

All somatic symptoms disappeared after a follow-up visit 2 months later.

**Lessons:**

This case provides valuable information to the clinic, through the compilation of literature and case analysis, the author found that paying attention to mental health, to women in poor areas and to women from families of low education level are indispensable in medical diagnosis and treatment.

## 1. Introduction

Ibuprofen Sustained-Release capsules are common nonsteroidal anti-inflammatory drugs, because of the easy access and high clinical efficiency, in poor areas of China, many families normally use backup ibuprofen to relieve fever, analgesia, anti-inflammatory, and to deal with pain and fever in any part of the body. In the previous literature, there were few cases of taking ibuprofen in large doses, and its adverse effects were mainly reported to include duodenal ulcer or perforation, lactic acidosis, and multiple organ system failure.^[1,2]^ This case reports a rare acute large gastric ulcer accompanied by transient hypertension after ingesting a large amount of ibuprofen, and this article intends to provide clinical data and learnings from experience to raise social awareness through case report.

## 2. Case presentation

A 35-year-old Chinese housewife took 48 Ibuprofen Sustained-Release capsules (300 mg/capsule) at 1 time to attract her husband’s attention and love. After 48 hours, she developed epigastric pain and her family took her to the hospital. The patient experienced severe tingling in the upper abdomen accompanied by increased eating, and dizziness, fatigue, heartburn, nausea, and retching also bothered her, and she had no previous related conditions.

**Figure 1.**
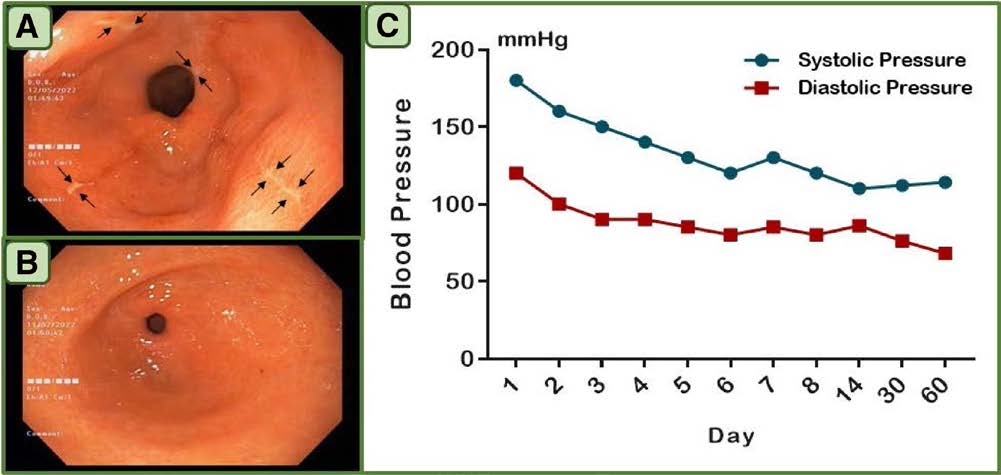
(A) Multiple linear deep concave ulcers, most of which were covered with thick white and yellow moss, mucosal hyperemia and edema around the ulcers, easy to bleed in the gastric antrum (gastroscope, under the white light). (B) The gastric antrum mucosa was white and mainly red, and its surface was smooth, without edema, erosion and bleeding. (C) The timeline of blood pressure progress.

Epigastric tenderness (+++) and elevated blood pressure (180/120 mm Hg) were found on physical examination, while the rest of the examination was not abnormal and there was no special family genetic history or the past history. Multiple linear deep concave ulcers appear in the antral region, most of the ulcers were covered with thick yellow and white moss, mucosal hyperemia and edema around the ulcer, easy to bleed to the touch, etc are detected in the gastroscopy (Fig. 1A). Laboratory tests found that the patient’s fecal occult blood and Helicobacter pylori were positive, hypersensitivity C-reactive protein was progressively elevated, and other laboratory tests, including liver and kidney function, were abnormal. At the same time, we used the Anxiety and Depression Scale to assess and found that she had 2 mental and psychological problems: moderate depression and mild cognitive impairment. Based on the above information, she was diagnosed with gastric antral ulcer (multiple stage A1), duodenocitis, chronic nonatrophic gastritis, Helicobacter pylori infection, moderate depression, and cognitive impairment. At the the early stage of treatment, fasting, water fasting, antihypertensive, acid suppression, gastric protection, and symptomatic treatment including anti-Helicobacter pylori were given. With the care of the family and after a series of treatments, the patients’ physical symptoms were significantly improved, and all clinical physical symptoms disappeared after 2 months of follow-up.

## 3. Discussion

This case occurred in a remote mountainous area in northern Shaanxi, China, due to the limitation of cultural level and medical knowledge, the patient’s understanding of drugs was insufficient, and the patient’s emotional dependence was very severe, which led to adverse events. With the deepening of medical assistance, and the timely visit to the doctor, the diagnosis was confirmed by gastroscopy immediately after admission and timely treatment was given. Exploring the cause of the disease is the focus of this case, and the mental psychological evaluation found that this patient did have such a problem.

The cause of the patient’s onset is a question worth consideration. The conventional maximum dose of ibuprofen is 900 mg/day, and a case of 60,000 mg/day was previously reported to be a suicidal patient.^[3]^ The evaluation of the patient’s mental and psychological state showed that the patient had obvious depression with cognitive impairment, and took a large dose of drugs after being stimulated by the family, which led to the occurrence of this incident. Therefore, mental health remains an important concern for medication misuse.

The most common adverse reaction of the clinical routine dose of ibuprofen is gastrointestinal mucosal damage, especially the above gastrointestinal mucosal damage, The probability of gastrointestinal mucosal damage together with hypertension is not common, this patient has all the above symptoms at the same time. Acute gastric mucosal injury caused by high-dose oral ibuprofen has been reported less clinically, and gastric microbleeding and gastric blood flow velocity in the gastric mucosa under chronic stimulation of low-dose nonsteroidal anti-inflammatory drugs (NSAIDs) are common, which may be related to NSAIDs promoting platelet/neutrophil adhesion and neutrophil/endothelial cell interactions in the gastric mucosa.^[4]^ Related experimental zoological studies have shown that NSAIDs can stimulate acute gastric mucosal injury in animals and have shown elevated hypersensitivity C-reactive protein.^[5]^ Although the hypertension caused by ibuprofen may be related to ibuprofen by constricting tiny blood vessels,^[6]^ a slow increase in hypertension has been reported in the context of continuous ibuprofen use,^[7,8]^ rather than a sudden increase in this case (Fig. 1C), which may be related to drug overdose factors.

The greatest damage of NSAIDs to the gastric mucosa reported in the previous literature mostly occurred 24 hours after medication, and the damage of NSAIDs to the upper digestive mucosa existed in discrete form, and more commonly in duodenal bulb mucosal injury.^[9]^ In this case, gastric mucosal damage was found 48 hours after taking the drug, mainly manifested in multiple linear and deeply concave ulcers of the antrum as the main feature. The invasion factors of the patient were enhanced after taking an overdose of ibuprofen. The antral mucosa may be weakened due to excessive stimulation, and acute antral mucosal damage occurred, while the duodenal bulb only showed mild erosion. The reason is also worth further consideration.

As we all know, Helicobacter pylori infection and nonsteroidal anti-inflammatory drugs are common factors leading to gastric mucosal lesions. Respectively, this patient has no disease history and positive HP test after admission, but according to previous research data, it is currently controversial whether the 2 interact will cause gastric mucosal damage.^[10–12]^ Studies have shown that the clinical safety of ibuprofen is relatively high, and the occurrence of visceral injury is related to the dosage, duration, gender, age, and combined medication.^[13–15]^

Reports of damage to the gastric mucosa caused by drug overdose are not uncommon, but clinical cases of taking large doses of more than 10 times of the standard dose are rare. However, fortunately, the symptoms of gastric mucosal injury and hypertension disappeared quickly under the intervention of effective treatment measures, and no other adverse consequences were caused in the following follow-up (Fig. 1B). With the rapid development of China’s modern economy and the impact of social ideology and culture, low-educated people are trying to break through the shackles of traditional ideas and culture while trying to seek psychological and spiritual peace. Due to the lack of relevant knowledge and cognitive impairment, the harm caused by extreme behavior to the physical and mental health of housewives in poor areas is not only a medical problem, but also a social problem that requires in-depth consideration. While paying attention to the life and health of patients, doctors need to pay close attention to the spiritual world and mental health of patients to avoid the occurrence of secondary injuries.

## Author contributions

**Conceptualization:** Zhang Meijuan.

**Software:** Penglong Yu.

**Supervision:** Jie Yuan.

**Visualization:** Tao Yu.

**Writing – original draft:** Dan Sun.

**Writing – review & editing:** Dan Sun.
